# Lymphatic pumping technique in mice alters blood parameters and metastatic melanoma in an age-dependent manner

**DOI:** 10.3389/ebm.2026.10850

**Published:** 2026-03-30

**Authors:** Christopher Walsh, Matthew Kirstein, Elise Wagner, Emily Scott, Jerome Walsh, Shashank Reddy, Nathan Hoggard, Arshad Ahmad, Reetobrata Basu, Sam Mathes, Yanrong Qian, John J. Kopchick

**Affiliations:** 1 Heritage College of Osteopathic Medicine, Ohio University, Athens, OH, United States; 2 Institute for Molecular Medicine and Aging, Ohio University, Athens, OH, United States; 3 Department of Biological Sciences, Ohio University, Athens, OH, United States

**Keywords:** lymphatic pumping technique, manual therapy, melanoma, metastasis, osteopathy

## Abstract

Therapeutic touch applied to primary tumors can increase metastasis. The goal of this project was to determine whether touch applied to metastatic tumors also increases metastasis. We evaluated touch on a mouse model of experimental metastasis using a manual treatment called Lymphatic Pumping Technique (LPT), which increases lymphatic fluid flow and is contraindicated in patients with cancer. The LPT, or a sham treatment, was administered for 5 minutes while the mice were anesthetized with vaporized isoflurane. Young adult (3 months old) and aged (20–24 months old) mice received daily sham or LPT treatments for 7 days prior to the injection of 200k B16F10-luc2 mouse melanoma cells into the tail vein, then treated every other day for 21 days. In middle-aged (9–11 months old) mice, we waited 8 days after tumor injection to start treatments and assessed the effect of LPT on immunotherapy efficacy. These mice also received either LPT or sham every other day, along with four doses of 200 µg anti–PD-1 or isotype control antibody. LPT did not increase tumor growth or spread in any of the experiments. Surprisingly, LPT was negatively associated with metastasis in young and middle-aged mice, without enhancing or diminishing the efficacy of immunotherapy. In mice without cancer, LPT rapidly elevated red blood cell, white blood cell, and platelet counts in young, but not middle-aged, animals. Taken together, these findings suggest that therapeutic touch near metastatic tumors does not worsen disease and may confer an age-dependent benefit.

## Impact statement

Current clinical guidelines broadly discourage therapeutic touch near all tumors based on evidence derived almost exclusively from primary tumor models. This study is the first to directly evaluate the effect of touch on metastatic disease. Using mouse models of experimental metastasis and the lymphatic pumping technique, a form of touch that maximizes theoretical risk, we demonstrate that touch does not increase metastatic growth or spread and, in fact, reduces it in an age-dependent manner. These findings reveal that the biological impact of touch on cancer is not uniform but varies with age and disease stage. By showing that therapeutic touch can be safe and potentially beneficial in diffuse metastatic disease, this work challenges long-standing assumptions in oncology. Integrating osteopathic manual medicine with molecular cancer biology, this study bridges two traditionally separate fields and identifies a novel, non-pharmacologic avenue for adjunctive cancer therapy.

## Introduction

Metastatic tumors growing outside of the primary tumor are the leading cause of death in patients with cancer [1]. Surgical and radiation-based treatments are highly effective for localized tumors but become limited in metastatic disease, which can present with many tumors in multiple organs. Systemic chemical therapies remain the standard approach for the treatment of metastatic disease, yet survival declines precipitously after metastasis. For example, the 5-year survival rate after the diagnosis of melanoma drops from nearly 100% for localized disease to 35% for patients with tumors that have grown in distant organs [2]. These stark disparities underscore the importance of specifically studying and targeting metastatic disease.

An essential stage of cancer metastasis is colonization, during which newly arrived cancer cells remodel the local organ interstitium into a permissive tumor microenvironment [3]. An important driver of this transformation is the disruption of the organ’s fascial network. Fascia, a connective tissue continuum that organizes and mechanically integrates organs and their microenvironments, exerts biomechanical forces that influence cell behavior. When dysregulated, the fascia can promote tumorigenesis and metastatic progression [4]. Emerging work focused on manual techniques has introduced the concept that physical manipulation of the fascia could be used to study, and potentially disrupt, the tumor microenvironment [5].

Despite this potential, the effects of touch on metastatic disease remain largely unexplored. In contrast, physical manipulation near primary tumors has been associated with increased metastatic spread in both animal and large-scale observational studies [6–8]. These findings have shaped clinical guidelines, which broadly recommend against the use of manual therapies near any tumor [9]. While appropriately cautious, such recommendations are largely extrapolated from studies of primary tumors and may inadvertently limit beneficial supportive care. Therapeutic touch has demonstrated value in oncology [10], including reductions in cancer-associated pain [11], chemotherapy-induced constipation [12], and post mastectomy lymphedema [13]. Osteopathic manipulative medicine is a form of manual medicine capable of addressing cancer comorbidities, but patients with diffuse metastatic disease are often excluded due to a theoretical risk of promoting tumor spread.

One osteopathic technique of particular concern is the lymphatic pumping technique (LPT), which is a commonly used manipulation that increases lymph production and flow through rhythmic compression and release of tissue spaces. This mechanical force distorts the interstitium, opening primary lymphatic valves, and increasing perivascular lymphatic pressure, thereby enhancing lymph production and flow through the lymphatic system [14]. Because LPT increases tissue fluid movement, it is presumed to pose a heightened risk for facilitating cancer dissemination and is specifically contraindicated in patients with cancer [9]. For this reason, we believed LPT could be used to study the risk posed by physical manipulation of the metastatic tumor.

**FIGURE 1:**
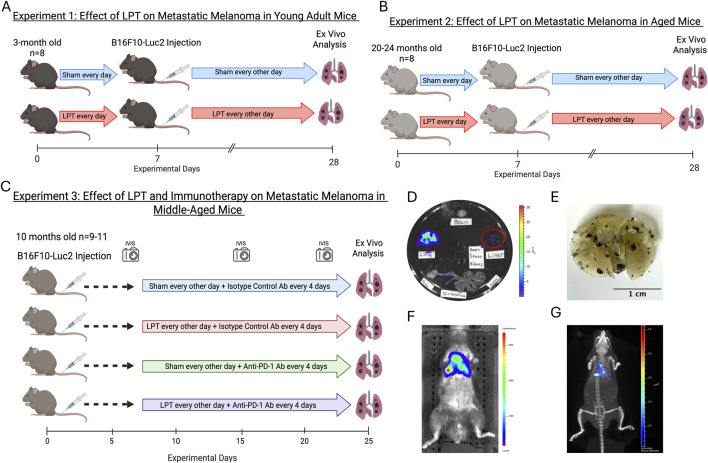
Methods and experimental design. An illustrated workflow for the first experiment with young adult mice **(A)**, the second experiment with aged mice **(B)**, and the third experiment with middle-aged mice and the addition of immunotherapy **(C)**. The lymphatic pumping technique (LPT) sequence used in the treatment group **(D)**, along with the hand placement **(G)** used during thoracic LPT. Example of the 2D bioluminescent imaging used to measure tumor burden *in vivo* **(H)** and *ex vivo* **(E)**. **(F)** A photo of formalin-fixed lungs from mice bearing B16F10-Fluc metastatic tumor growth. **(I)** Example of the 3D bioluminescent imaging of B16F10-Fluc lung metastasis. The graphics in A, B, and C were created in BioRender. Walsh, C. (2026) https://BioRender.com/m1cz62o.

We designed the experiments, detailed in Figure 1, to assess the effect of LPT on melanoma growth and spread in a mouse model of experimental metastasis using young adult and aged mice. Ageing increases tissue stiffness [15] and the accumulation of dysregulated immune mechanisms [16]. Since LPT relies on tissue distortion to activate the lymphatic system, we hypothesized that the stiffer tissue in older animals would be less responsive to LPT. Likewise, the depressed capacity of the aged immune system to mount a successful immune response could limit any immune benefit brought by LPT in the aged cohort. Following the observation of a beneficial impact of LPT in young mice, we investigated whether LPT could further enhance the anti-tumor immune response induced by immune checkpoint blockade in middle-aged mice. In addition to *in vivo* and *ex vivo* measures of tumor burden, we assessed the effect of LPT on the tumor microenvironment by measuring the expression of proteins associated with epithelial-to-mesenchymal transition and lymphangiogenesis. Lastly, we evaluated the influence of age on LPT’s ability to move non-cancerous cells by measuring the complete blood cell count in whole blood collected from mice before and immediately after LPT.

## Materials and methods

### Lymphatic pumping technique (LPT)

The LPT used in this study is a series of manual therapies designed to increase whole-body lymphatic flow by relaxing myofascial tension at transition areas around the thorax and abdomen, followed by lymphatic pumping of the thoracic and abdominal cavities. Similar thoracic and abdominal LPTs have been used separately in preclinical rodent models of pneumonia [17] and inflammatory bowel disease [18]. The LPT and sham treatments were performed under general anesthesia using 2.0–2.5% isoflurane and prewarmed heating pads. Mice in the control group received a sham treatment consisting of 5 minutes of general anesthesia only. Mice in the LPT group received the following series of manual therapies: a 1-min soft-tissue massage, 2 min of thoracic LPT, and 2 min of abdominal LPT. To allow time for normal respirations, the mouse was not touched for 15 seconds between the rounds of thoracic LPT. The soft tissue massage consisted of medial-to-lateral kneading of the diaphragm and circular massaging of the axilla and inguinal areas. Thoracic lymphatic pumping was performed by placing the thumb on one side of the rib cage and an index and middle finger on the opposite rib cage, specifically, 1 cm lateral and cranial to the xiphoid cartilage. Then, with the index finger of the opposite hand, light pressure is exerted over the sternum in a dorsal-caudal direction until rib cage compression is appreciated and then released. Similarly, abdominal lymphatic pumping was performed by placing the thumb and forefinger on the lateral edge of the abdomen, then compressing the abdomen with the finger of the opposite hand in a dorsal-cranial direction until counterpressure from the ribs against the diaphragm was appreciated. This pressure is applied at a rate of 1 compression per second for both techniques. The same 5-min LPT sequence was used for all experiments in this study. A video of the LPT treatment is available in Supplementary Video S1.

### Melanoma lung metastasis model

A widely used method for studying metastatic tumors is the experimental lung metastasis model, which mimics the metastatic process by injecting cancer cells into the mouse’s tail vein. The cells used were B16F10-Fluc-Puro mouse melanoma cells (Imanis Cat# CL052, RRID:CVCL_QZ90), which were maintained in T75 flasks with regular media changes consisting of Dulbecco’s modified Eagle’s medium containing 10% fetal bovine serum, 1% Penicillin/Streptomycin, and 1ug/mL puromycin and injected into the mice before cell passage 9. Luciferase expression was checked in the cell passage before injection by plating cells at increasing concentrations on a 12-well plate. The following day, the cells were washed with warm phosphate-buffered saline (PBS) and incubated in PBS containing 15 µg/mL of IVISbrite D-Luciferin (#122799, Revity), then imaged with an IVIS imaging unit. On the day of tumor injection, cells from the same passage number were washed twice, dissociated from their flask using 0.25% trypsin-EDTA, centrifuged, resuspended in cold PBS at 2 million cells/mL, and kept on ice until tail vein injection. Before injection, mice were warmed under a heat lamp for 1 min and then placed under general anesthesia as previously described. The injection location over the lateral tail vein was wiped with 70% alcohol, then punctured with a 29 G needle to intravenously inject 200,000 melanoma cells in 100 μL of PBS. The number of cells was selected based on a standard protocol, which states that metastatic tumors can be measured for 3 weeks after cancer cell injection and before death is expected due to pulmonary congestion [19].

Wild-type male C57BL/6J mice were used in all cancer experiments (RRID:IMSR_JAX:000664). We used 3-month-old mice for the young group, 10-month-old mice for the middle-aged group, and 20–24-month-old mice for the aged group, roughly corresponding to humans aged 20, 45, and 80 years, respectively [20]. Mice were randomly assigned to groups before the experiment, and each cage received the same treatment. If a mouse died before the experimental endpoint, the animal was removed from the study. Only one mouse died early and was removed from the study: a middle-aged mouse in the control group.

**FIGURE 2:**
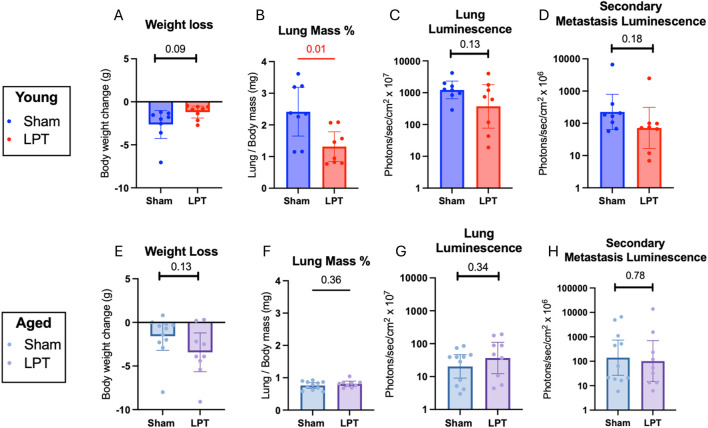
Increased lymphatic flow before and after metastasis from LPT did not increase tumor growth or spread in young adult or aged animals. Measures of tumor burden from the first two experiments with young adult and aged mice. The body weight changes for each young **(A)** and aged **(E)** mouse. The change in the percentage of body mass contributed to by the lung in the young **(B)** and aged cohorts **(F)**. *Ex vivo* luminescence in the primary site of metastasis, the lung, of the young **(C)** and aged **(G)** animals, and the secondary metastatic sites, including the brain, heart, spleen, kidneys, liver, and gastrointestinal tract of the young **(D)** and aged **(H)** animals. The statistical tests used were unpaired t-tests **(B,F)**, Welch’s t-test in **(C,D,G,H)**, Mann-Whitney U-test **(E)**, and the KS test **(A)**. Bar graphs show the mean **(A,B,E,F)** or the geometric mean **(C,D,G,H)** with 95% CI. A red line and a number signify a significant change (p-value ≤0.05).

The experimental design of the four *in vivo* experiments reported in this study is shown in Figure 1. The goals of Experiments 1 (Figure 1A) and 2 (Figure 1B) were to assess how increased lymphatic flow affects tumor metastasis at both young adult and advanced ages. Therefore, we performed LPT before and after the injection of cancer cells. Specifically, the sham and LPT treatments were administered daily on days 1–7; the cancer cells were injected on day 8; and the animals were treated every other day on days 7–27. On day 28, mice were euthanized with carbon dioxide inhalation. In Experiment 3 (Figure 1C), we aimed to increase the clinical translatability of the study by initiating LPT after cancer cell injection and combining it with a commonly used treatment for metastatic melanoma, an immune checkpoint inhibitor. In addition to the sham and LPT treatments, the mice were treated with anti-mouse programmed cell death protein 1 (PD1) antibody (#PA007163.m2cLA, Syd Labs) or an isotype control antibody (#PA007141, Syd Labs). Four treatments of 200 μg of antibody diluted in 100 μL of PBS (2 mg/mL) were administered intraperitoneally between days 9 and 21 of the experiment. The anti-PD1 dosing regimen of 200 ug per injection was selected based on established murine immunotherapy protocols rather than allometric scaling from human doses, consistent with prior studies demonstrating biological efficacy at this dose [21, 22]. Mice were divided into four treatment groups: group 1 received sham treatment and isotype control antibodies; group 2 received LPT and isotype control antibodies; group 3 received sham treatment and anti-PD1 antibodies; and group 4 received LPT treatment and anti-PD1 antibodies. The cancer cells were injected on experimental day 0, treatments started on day 8, ended on day 22, and the mice were euthanized with carbon dioxide inhalation and on day 25.

At the end of each experiment, ex vivo bioluminescent imaging was performed to quantify tumor growth and spread. Prior to euthanasia, mice were injected with D-luciferin (#88292, Thermo Fisher Scientific) at 150 mg/kg of body weight, dissolved in PBS, into the peritoneum. After 7 min, the mice were euthanized by carbon dioxide inhalation, and the organs (brain, lungs, heart, spleen, kidneys, liver, pancreas, and small and large intestines) were removed, placed on a plastic dish, and immediately imaged. An ROI was drawn around each individual organ to calculate the total signal for each mouse. Figure 1 depicts examples of ex vivo imaging (Figure 1D), formalin-fixed lungs (Figure 1E), 2D imaging (Figure 1F), 3D imaging (Figure 1G) generated using the techniques described above. For experiments 1 and 2, tumors were imaged using the Xenogen IVIS 100 Imaging System (RRID:SCR_020901), and the images were analyzed with Living Image 3.50 software (RRID:SCR_014247). A new IVIS imaging unit (IVIS SpectrumCT 2, #CLS158737, Revvity) was used for Experiment 3, offering improved tumor visualization and a standardized alternative method for calculating the bioluminescent signal, called counts, using Living Image 4.8 Software (Revvity). During weekly in vivo imaging sessions, mice were intraperitoneally injected with 4.5 mg of diluted IVISbrite D-Luciferin (#122799, Revvity) in 150 μL and imaged using auto-exposure settings, with 2D and 3D CT-assisted image reconstruction.

### Saphenous bleeding and CBC

By collecting whole blood, we were able to assess the ability of LPT to flux non-cancerous cells into the bloodstream. For this, we used young adult and middle-aged male and female mice. They were warmed under a heat lamp for 1 min and then placed under general anesthesia using vaporized isoflurane as described previously. The skin above the lateral saphenous vein was shaved, wiped with 70% ethanol, and coated with a small amount of petroleum jelly. The vein was pierced with a 27-gauge needle, and 100 μL of blood was collected into an EDTA-coated polypropylene blood collection tube (Microvette, Sarstedt). After achieving hemostasis using finger pressure, 5 minutes of sham or LPT treatment were performed. After the intervention, a subsequent blood sample was collected from the opposite leg. After blood collection, each sample was placed on a blood rocker for 10 min and then analyzed using an automated hematology system (Hemavet 950FS, Drew Scientific, USA). For each mouse, the blood parameters before LPT were subtracted from those after LPT. A two-tailed t-test was then performed for each parameter. The blood parameters measured were white blood cells, neutrophils, lymphocytes, monocytes, eosinophils, basophils, red blood cells, hemoglobin, hematocrit, mean corpuscular volume, mean corpuscular hemoglobin, mean corpuscular hemoglobin concentration, red blood cell distribution width, and platelet count.

### Western blot analysis

After dissection, the heart and lungs were snap frozen in liquid nitrogen. Proteins were extracted from 35 mg of tissues using a 2x RIPA protein extraction buffer with 1:100 protease/phosphatase inhibitor and 1:100 phenylmethylsulfonyl fluoride. A Precellys tissue homogenizer was used to create a lysate, which was then centrifuged and sonicated at 4 °C for 15 min to purify the protein. Protein concentrations were determined using the Bradford assay, allowing for the separation of 50 μg of each sample via SDS-PAGE. Proteins were transferred from the gel to PVDF membranes using a Trans-Blot Turbo Transfer System (BIO-RAD). Total protein transferred to the blot was measured using AdvanStain Ponceau protein staining (#R-03021-D50, Advansta). Membranes were blotted overnight with the following primary antibodies against lymphatic vessel hyaluronic receptor 1(LYVE1) (Goat, 1:1000, R and D Systems Cat# AF2125, RRID:AB_2297188), podoplanin (PDPN) (Rat, 1:1000, Abcam Cat# ab256559, RRID:AB_2936436), prospero homeodomain protein 1 (PROX1) (Rabbit, 1:1000, Abcam Cat# ab199359, RRID:AB_2868427), vascular endothelial growth factor receptor 3 (VEGFR3) (Rat, 1:1000, Thermo Fisher Scientific Cat# 14–5988-82, RRID:AB_467795), platelet endothelial cell adhesion molecule (CD31) (Rabbit, 1:500, Proteintech Cat# 28083-1-AP, RRID:AB_2881055), E-cadherin (ECAD) (Rabbit, 1:1000, Cell Signaling Technology Cat# 3195, RRID:AB_2291471), N-cadherin (NCAD) (Rabbit, 1:1000, Cell Signaling Technology Cat# 13116, RRID:AB_2687616), vimentin (VIM) (Rabbit, 1:1000, Cell Signaling Technology Cat# 5741, RRID:AB_10695459), slug (Rabit, 1:1000, Cell Signaling Technology Cat# 9585, RRID:AB_2239535), programmed death ligand 1 PDL1(Rabbit, 1:1000, Cell Signaling Technology Cat# 60475, RRID:AB_2924680) and programmed death ligand 2 (PDL2) (Rabbit, 1:1000, Cell Signaling Technology Cat# 82723, RRID:AB_2799999). After multiple washes, the blots were incubated for 1 hour with secondary antibodies linked to horseradish peroxidase (HRP), including an anti-rat-HRP (Goat, 1:2000, Thermo Fisher Scientific Cat# 31470, RRID:AB_228356), an anti-goat-HRP (Chicken, 1:2000, R and D Systems Cat# HAF019, RRID:AB_573132), and an anti-rabbit-HRP (Goat, 1:2000, Cell Signaling Technology Cat# 7074, RRID:AB_2099233). Blots were developed using luminol-based enhanced chemiluminescence HRP-substrate (SuperSignal West Dura Extended Duration Substrate, ThermoFisher # 34075) and imaged on an Azure 300. Densitometric analysis was performed using Image Studio. The signal of the target proteins was normalized to the total protein stain in that sample’s lane by dividing the target signal by the normalized total protein signal.

### Quantitative polymerase chain reaction (qPCR)

Total RNA was extracted from 50 mg of snap-frozen tissue using the GeneJET RNA Purification Kit (Thermo Scientific # K0731) according to the manufacturer’s protocols. Equal amounts of total RNA, quantified with nanodrop, were then converted to cDNA using oligo-dT reverse transcription with the High-Capacity cDNA Reverse Transcription Kit (Thermo Scientific # 4368814). Quantitative PCR was performed using SYBR Green dye-based amplification and detection with SYBR Green Universal Master Mix (Applied Biosystems # 4309155) and a QuantStudio Real-Time PCR System 3 (Applied Biosystems). Target gene expression was normalized to actin and prostaglandin E receptor 2 expression and compared between treatment groups using the ΔΔ CT method. The KiCqStart primer (Millipore Sigma # KSPQ12012) sequence pairs used were for mouse prospero homeobox 1 (*Prox1*; Fwd: GAC​GTG​AAG​TTC​AAC​AGA​TG and Rev: TTG​TTG​TAG​TGC​ATG​TTG​AG), vascular endothelial growth factor receptor 3 (*Flt4*; Fwd: AGC​TCT​ACA​TAT​CAC​CGA​AG and Rev: CAC​AGT​TGT​AAT​ATC​TGG​CTG), podoplanin (*Pdpn*; Fwd: AGATAAGAAAGATGGCTTGC and Rev: AACAACAATGAAGATCCCTC), lymphatic vessel hyaluronic acid receptor 1 (*Lyve1*; Fwd: ACG​TGA​AAA​GGT​ATG​TGA​AG and Rev: CTC​CTC​TGG​GTT​TTT​AAT​GG), collagen 1 (*Col1a1*; Fwd: CAG​CGA​TTA​CTA​CTG​GAT​TG and Rev: GAT​AGT​CTC​TCC​TAA​CCA​GAC), collagen 3 (*Col3a1*; Fwd: AAC​ATG​TTT​CTT​CTC​TGC​AC and Rev: ACT​CAA​GAG​TGG​AGA​ATA​CTG), collagen 4 (*Col4a1*; Fwd: CGG​ATA​TTC​ATT​CCT​CAT​GC and Rev: CAG​AAG​CTG​TAC​TTG​TTA​GC), collagen 18 (*Col18*; Fwd: GTA​GAT​TCT​ATA​GGA​GCT​GAG​AC and Rev: CTC​CCT​TTT​GTC​CTT​TCA​TAC), growth hormone (*Gh1*; Fwd: TCCAGTCTGTTTTCTAATGC and Rev: TCGAACTCTTTGTAGGTGTC), growth hormone receptor (*Ghr*; Fwd: ACTGTCCAGTGTACTCATTG and Rev: CTGGATATCTTCTTCACATGC), insulin-like growth factor receptor 1 (*Igf1r*; Fwd: AGA​ACC​GAA​TCA​TCA​TAA​CG and Rev: TTT​TAA​ATG​GTG​CCT​CCT​TG), firefly luciferase (*luc2*; Fwd: CAC​CGT​CGT​ATT​CGT​GAG​CA and Rev: AGT​CGT​ACT​CGT​TGA​AGC​CG), prostaglandin E receptor 2 (*Ptger2*; Fwd: CCT​GCT​GCT​TAT​CGT​GGC​TG and Rev: GCC​AGG​AGA​ATG​AGG​TGG​TC).

### Statistics and data representation

The bioluminescent signal used to measure tumor burden in this study follows a lognormal distribution; therefore, geometric means were compared between groups, and the bioluminescent data were graphed on a log10 scale. The standard mean and linear scale were used to represent all other data. Error bars were expressed as the mean with the 95% confidence interval (CI) for error bars. The statistical test used to compare normal data was represented by a straight line, while a capped line indicated a comparison with nonparametric data or groups with unequal variances.

For each statistical comparison, we assessed normality and variance between biological groups using the Shapiro-Wilk and F-test for two-group comparisons and the Shapiro-Wilk and Brown-Forsythe tests for comparisons among multiple groups [23]. For comparisons between two independent groups, normally distributed data with homogeneous variances were analyzed with an unpaired two-tailed t-test, whereas normally distributed data with heterogeneous variances were analyzed with Welch’s t-test, non-parametric data with equal variances were evaluated with the Mann–Whitney U test, and non-parametric data with unequal variances were examined with the Kolmogorov–Smirnov (KS) test. For comparisons among more than two groups, normally distributed data with equal variances were assessed by ordinary one-way ANOVA with the Tukey-Kramer *post hoc* test and displayed with capitalized compact letter display; non-parametric data, irrespective of variance homogeneity, were analyzed with the Kruskal–Wallis test with Dunn’s *post hoc* analysis and displayed with lower-case compact letter display. For the ordinary two-way ANOVAs, the full model was used to assess the effects of age, treatment, and the interaction between age and treatment on the blood cell parameters. If the primary analysis indicated statistical significance, the Bonferroni *post hoc* test was then performed, and its p-value was displayed. P-values from each test were displayed on the corresponding graphs, colored red when statistically significant (p ≤ 0.05) and black when non-significant (p > 0.05). Graphical data and statistical analysis were performed using the statistical software Prism 10 (GraphPad Prism, RRID:SCR_002798).

## Results

### LPT before and after metastasis did not increase tumor growth or spread in young or aged animals

The impact of LPT on metastatic disease was assessed using four markers of tumor burden in old and young adult mice: weight loss, lung mass percent, lung luminescence, and secondary organ luminescence (Figure 2). LPT did not significantly increase tumor burden in young adult or old mice. Lung mass percent is a normalized measure of lung weight and a historical gross measure of tumor burden. In young animals that received LPT before and after the IV injection of cancer cells, lung mass percentage decreased significantly (p = 0.01). The luminescence from luciferase activity in cancer cells was quantified as a specific measure of tumor burden. LPT did not statistically affect luminescence in the lungs or organs outside of the lungs. Although this was true for both age groups, a trend of decreased luminescence in the lungs and secondary organs was observed in the younger animals that received LPT, with p-values of 0.13 and 0.18, respectively. LPT did not significantly affect weight loss in young adult or aged mice.

### LPT altered gene and protein expression in the lung tumor microenvironment

**FIGURE 3:**
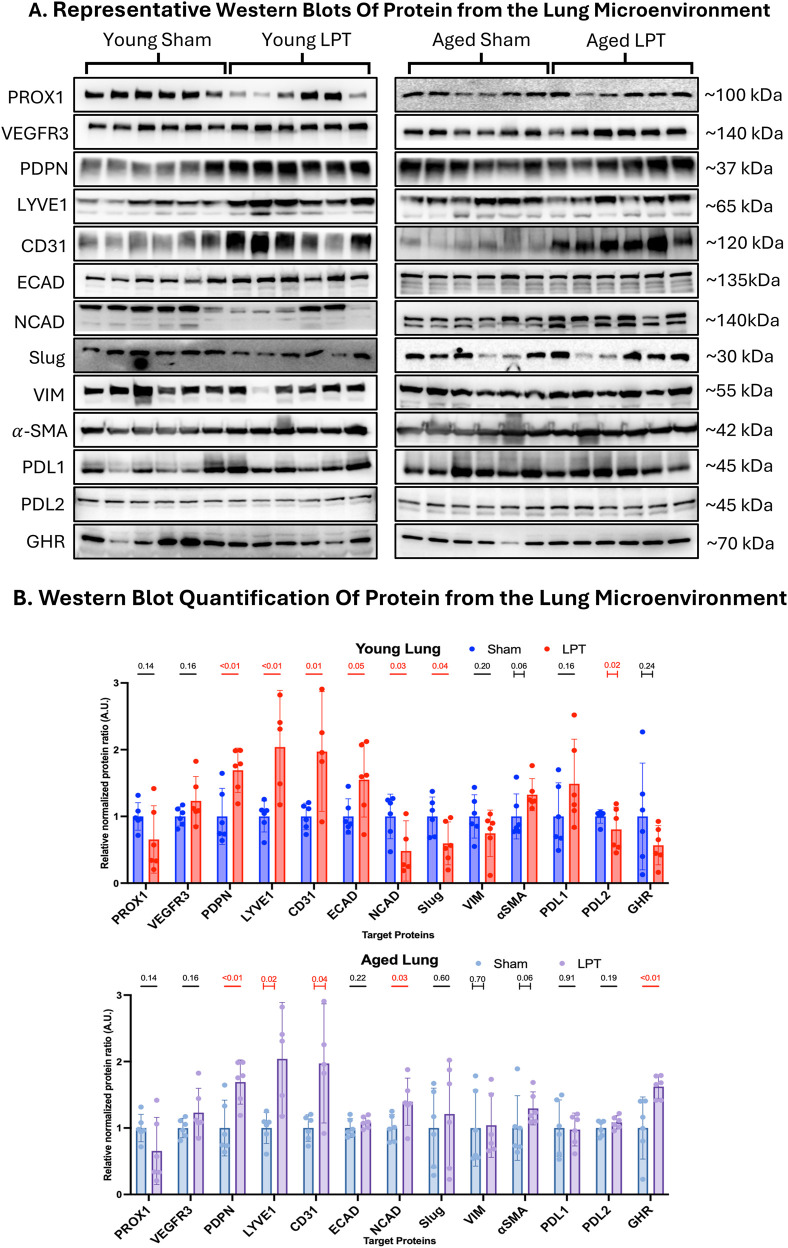
LPT altered gene expression in the lung microenvironment **(A)** Western blotting was performed on 50 mg of lung tissue taken at the time of dissection for numerous target proteins. **(B)** The expression of these targets was normalized to the total protein stain and then graphed as fold change. The unpaired t-test was used for all comparisons, except for the young α SMA, aged α SMA, and aged VIM, which used the Mann-Whitney U-test; the young PDL2, young GHR, and aged LYVE1, which used Welch’s t-test; and the young CD31 and aged CD31, which used the KS test. Bar graphs show the mean with the 95% CI. A red line and a number signify a significant change (p-value ≤0.05), and a capped line below the p-value indicates the use of a non-parametric test.

The following proteins of interest (Figure 3) were selected to investigate three physiological events linked to (1) lymphangiogenesis (PROX1, VEGFR3, PDPN, LYVE1, CD31), (2) endothelial-to-mesenchymal transition (ECAD, NCAD, Slug, VIM, α -SMA, and (3) immune modulation (PDL1, PDL2, GHR). The most robust overlapping effect of LPT across the two ages was its impact on markers of lymphatic vessels. LPT nearly doubled the expression of CD31 (p = 0.01 and p = 0.04), LYVE1 (p < 0.01 and p = 0.02), and PDPN (p < 0.01 and p < 0.01) in both young adult and aged mice, respectively. The effect of LPT on markers of epithelial-to-mesenchymal transition was more pronounced in young animals. LPT increased the expression of the epithelial marker ECAD (p = 0.05) and decreased the expression of the pro-metastatic marker NCAD (p = 0.03) and Slug (p = 0.04) in the young animals. Only the pro-metastatic marker NCAD (p = 0.03) significantly changed in the aged animals after LPT treatment. Regarding immune modulation, LPT significantly decreased PDL2 expression (p = 0.02) in young adult mice and increased GHR expression (p < 0.01) in old mice.

**FIGURE 4:**
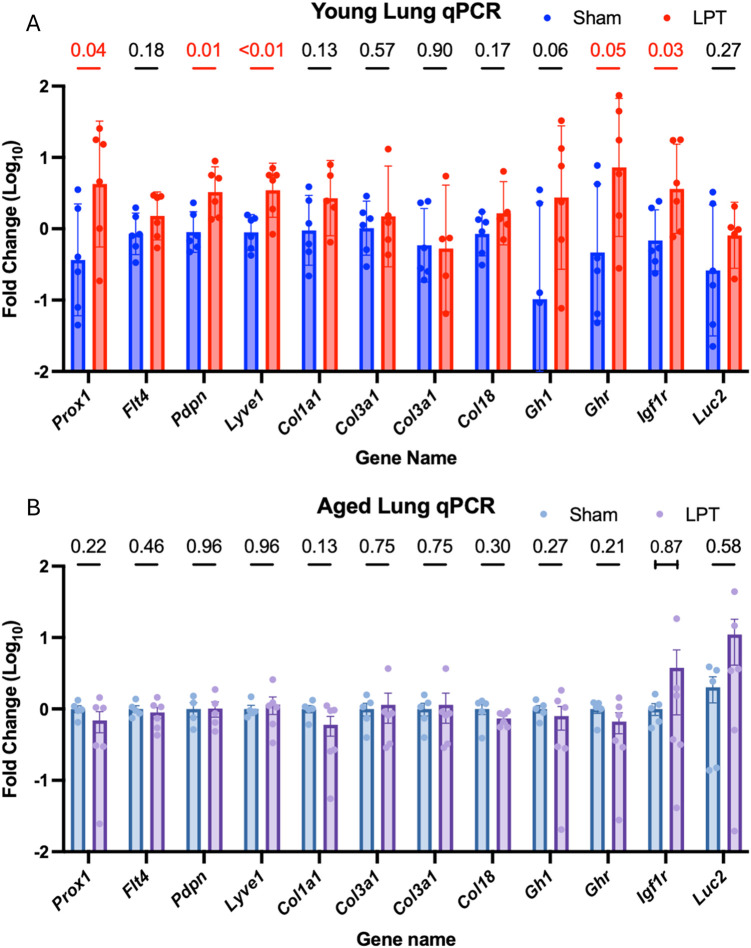
LPT altered the expression of mRNA in young but not aged lungs qPCR was performed on target genes in the lungs of young **(A)** and aged **(B)** animals, with results displayed as fold change, and bar graphs representing the geometric mean fold change (95% CI). A red line and a number signify a statistical change (p-value ≤0.05). The capped line below the p-value indicates the use of a non-parametric test.

Using qPCR to measure gene expression changes at the RNA level (Figure 4), we found that young adult animals receiving LPT had increased expression of the lymphangiogenesis-related RNA transcripts like *Lyve1*, *Flt4* (podoplanin), and *Prox1* compared to the sham treatment (p < 0.01, p < 0.01, p = 0.04, respectively). However, this effect was not observed in the aged group. Similarly, in the young cohort, there was an increased expression of *Ghr* (p = 0.05) and *Igf1r* (p = 0.03), but no significant change was observed in the aged cohort.

### LPT did not alter LEC protein expression in the liver microenvironment

**FIGURE 5:**
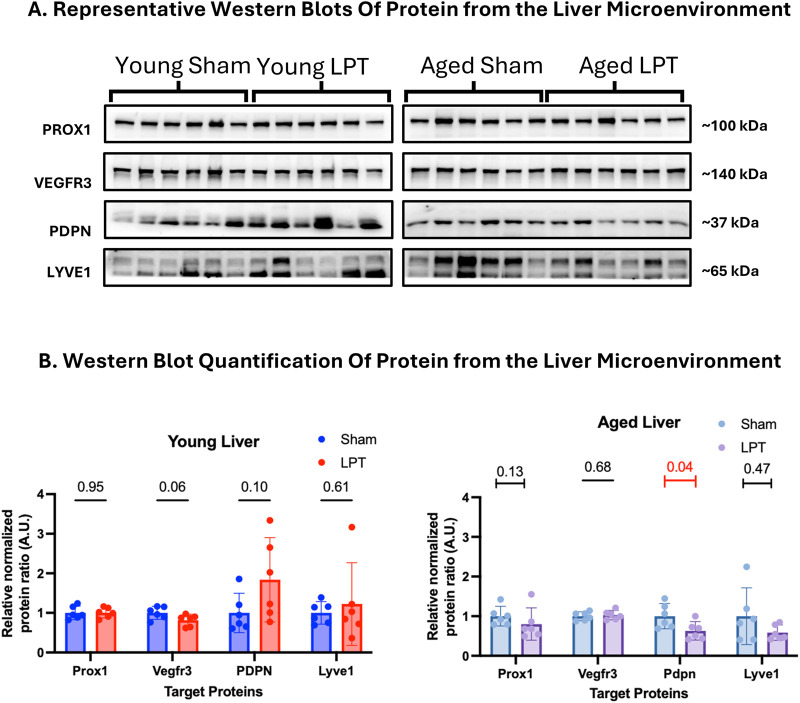
LPT did not alter protein expression in the liver microenvironment. Western blotting was performed on 50 mg of liver tissue taken at the time of dissection for lymphatic endothelial cell-specific protein expression **(A)**. The expression of these targets was normalized to the total protein stain and graphed as fold change **(B)**. The unpaired t-test was used for all comparisons aside from aged PROX1, which used the Mann-Whitney U-test; and the young LYVE1 and aged LYVE1, which used the KS test. The bar graphs represent the mean fold change with the 95% CI. A red line and a number signify a statistical change (p-value ≤0.05).

As with the lungs, liver protein samples were analyzed by western blot for expression of markers of the lymphatic system (Figure 5). LPT appeared to have little effect on the expression of LEC markers in both young and old animals. The only significant change caused by LPT was decreased PDPN expression in aged animals (p = 0.04).

### LPT reduced tumor burden in middle-aged mice but did not enhance immunotherapy

**FIGURE 6:**
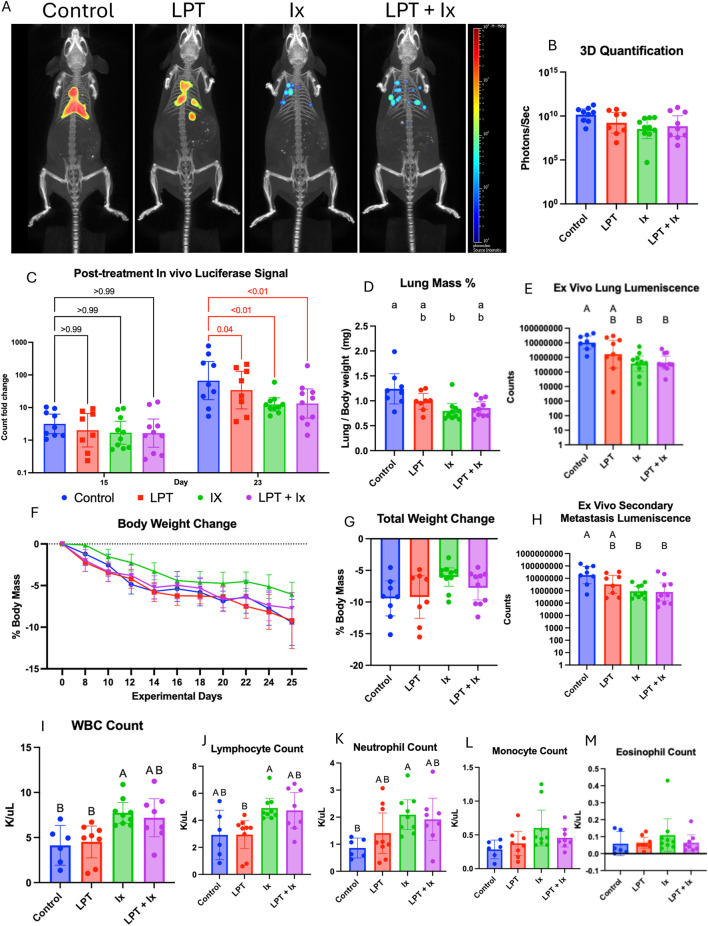
LPT reduced tumor burden in middle-aged mice and did not enhance or diminish the effect of immunotherapy. **(A)** Representative 3D bioluminescent images from each treatment group, taken 23 days after the injection of cancer cells. **(B)** Quantification of 3D bioluminescent images taken on day 23. **(C)** Quantified 2D whole-body luminescence on days 15 and 23, normalized to day 7, before treatment started. The statistical difference between the control and each treatment group was evaluated using a two-way ANOVA with Dunnett’s multiple comparisons test, and the results are indicated by a numeric p-value, which is highlighted in red if significant. **(D)** Statistical difference in lung mass percent was assessed with the Kruskal-Wallis test and *post hoc* analysis with Dunn’s multiple comparisons test. Differences in *ex vivo* luminescence in the lung **(E)** and secondary metastatic sites **(H)**, including the brain, heart, spleen, kidneys, liver, and gastrointestinal tract, were assessed using a lognormal ordinary ANOVA with Tukey’s multiple comparisons test. The mean percent change in each group’s body weight throughout the experiment **(F)** and the final percent weight change **(G)**, which showed no significant difference between the groups when assessed with a one-way ANOVA. Whole blood was collected during the dissection, parameters were analyzed using a Hemavet 950FS, and the difference between groups was assessed with an ordinary one-way ANOVA and *post hoc* analysis with Tukey’s multiple comparison test **(I–M)**. If the primary ANOVA analysis did not show a significant difference, the *post hoc* analysis to assess group differences was not performed, and the compact letter display was not created. Bar graphs show the mean **(D,F,G,I–M)** or the geometric mean **(B,C,E,H)** with 95% CI.

The therapeutic effect of LPT was assessed in Experiment 3, which compared middle-aged mice from four treatment groups that received a sham treatment and isotype control antibodies (Control), LPT and isotype control antibodies (LPT), sham treatment and immunotherapy (Ix), and mice that received LPT and immunotherapy (LPT + Ix). Two-dimensional and three-dimensional bioluminescent images were captured at multiple time points, as shown in Figure 6.

To control for variability in tumor cells injected intravenously, two-dimensional whole-body luminescence was measured on day 7, the day before treatment started, and again on days 15 and 23 after treatment started. The pre-treatment signal on day 7 was used to normalize the post-treatment signals on days 15 and 23 for the individual mouse. Compared to the control, the LPT group (p = 0.04), the Ix group (p < 0.01), and the LPT + Ix group (p < 0.01) all had significantly reduced *in vivo* luminescence on day 23. On day 25, *ex vivo* measures of tumor burden, including lung mass percentage, *ex vivo* lung luminescence, and *ex vivo* secondary organ luminescence, were collected, resulting in a bimodal distribution of tumor burden between mice treated with immunotherapy and those that were not. LPT did not interfere with the efficacy of immunotherapy in any of the measures.

To assess whether there were any metabolic effects, we tracked body weight throughout the experiment and changes in body composition parameters on day −1 (the day before cancer injection) and on day 24 (the day before dissection). LPT had little effect on weight loss and body composition. There was an insignificant trend that animals receiving immunotherapy had less weight loss. Among the mice receiving immunotherapy, those also receiving LPT showed greater fat loss (Supplementary Figure S1).

To assess immune-mediated effects, we measured the white blood cell count and differential on day 25, the day of dissection. Animals receiving immunotherapy had higher white blood cell counts than those that did not, primarily driven by increases in neutrophils and lymphocytes, with no significant change in monocyte, eosinophil, or basophil counts.

### LPT immediately and significantly increased the white blood cell, red blood cell, and platelet counts in whole blood in young but not in middle-aged animals

**FIGURE 7:**
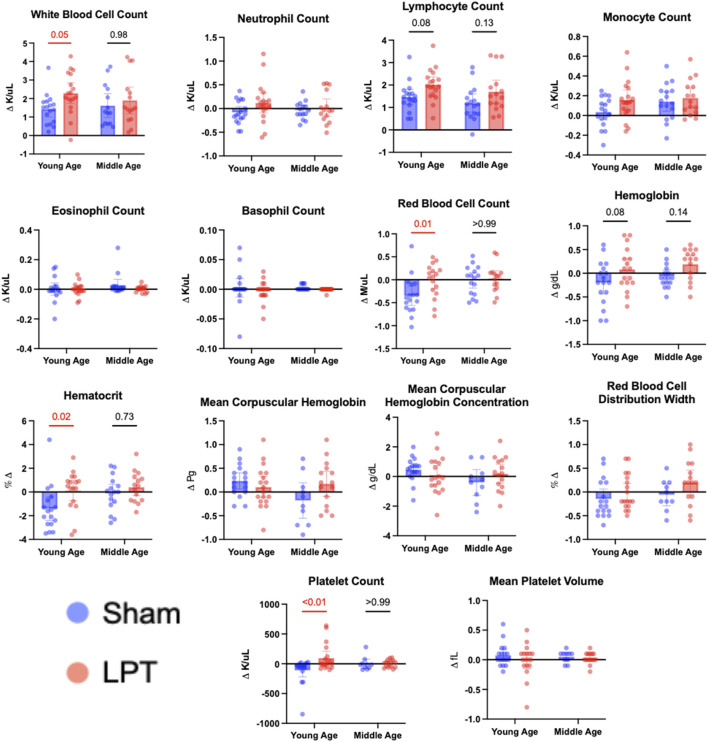
The effect of a 5-min LPT sequence on whole blood parameters in young adult and middle-aged mice. Whole blood collected immediately before and after a 5-min LPT sequence or sham treatment was analyzed using a Hemavet 950FS. The pre-treatment blood parameter was subtracted from the post-treatment parameter. The individual changes and the means of each group are represented as bar graphs with 95% CI. A statistical difference between the sham and LPT groups was assessed for both young and middle-aged animals using an ordinary two-way ANOVA, followed by *post hoc* analysis with Bonferroni’s multiple comparisons test. If the treatment significantly contributed to the observed variation, a *post hoc* analysis was conducted, and the p-values were shown on the graphs. A red line and a number signify a significant change (p-value ≤0.05).

To assess whether LPT can flux cells into or out of the circulatory system, we measured whole-blood parameters before and immediately after LPT. Figure 7 depicts the change in each blood parameter that was measured for young adult (3-month-old) and middle-aged (11-month-old) mice of both sexes (n = 16–18). Using a two-way ANOVA, it was found that LPT significantly increased red blood cell, white blood cell, platelet, and lymphocyte counts, as well as hemoglobin and hematocrit in whole blood. In the *post hoc* analysis, only the young animals’ blood parameters changed significantly. LPT elicited a robust change in blood parameters in the young animals; for example, white blood cells, red blood cells, and platelets increased by 64%, 96%, and 53%, respectively.

## Discussion

The primary motivation for this study was to assess the risks associated with physical manipulation in metastatic disease. Or simply, *is it dangerous to touch metastatic tumors*? Because tissue compression increases interstitial and lymphatic flow [24], touch-based therapies are often presumed to carry the potential to promote tumor dissemination. To test this assumption, we employed a mouse model of experimental metastasis and intentionally accentuated mechanical stimulation using LPT. Across all experiments, repeated touch did not increase the growth or spread of metastatic melanoma. Notably, in the young and middle-aged animals, LPT was associated with a reduction in metastatic burden, directly contradicting predictions derived from studies of primary tumors. This protective effect was supported by protein expression profiles within the tumor microenvironment, which revealed LPT-induced shifts towards an anti-metastatic phenotype in young adult mice. In middle-aged mice, both LPT and immune checkpoint antagonism reduced tumor burden independently, whereas their combination conferred neither additive benefit nor detriment. Together, these findings indicate that mechanical touch influences metastatic disease in a manner distinct from primary tumors and may even confer a therapeutic benefit that wanes with advanced age.

We identified a robust effect of LPT on whole blood parameters in young, but not middle-aged, adult mice. By performing a complete blood count and differential before and immediately after 5 min of LPT, we could detect immediate changes in whole-blood parameters. We discovered that LPT increases the number of red blood cells, white blood cells, and platelets in the blood; however, this effect is lost with increasing age. The immediate rise in parameters immediately after LPT suggests that LPT mobilizes pre-existing cells residing outside the systemic circulation. This is supported by previous studies showing that abdominal LPT mobilizes T cells from the gut-associated lymphoid tissue [25]. Therefore, the source of the white blood cells in this LPT sequence could be lymphoid tissue in the abdominal cavity and the thorax. The LPT sequence also puts direct repeated pressure on the spleen, making it the likely source of red blood cells and platelets. Two rationales for why LPT did not increase blood parameters in middle-aged animals could be that their extravascular reserve of these blood components is lower, or that increased tissue stiffness reduces LPT’s ability to flux cells. These preclinical results align with human data showing that LPT increases white blood cell counts in young, healthy males [26], while decreasing them in nursing home residents [27].

The only non-age-dependent result we found during this investigation was that LPT increased the expression of lymphatic-specific markers in the lung tumor microenvironment. This novel finding suggests that LPT either increases tumor-associated lymphangiogenesis or causes lung lymphangiogenesis. This is a unique presentation because tumor-induced lymphangiogenesis is often considered a negative prognostic factor. However, it has also been found that lymphatic endothelial cells can promote an anti-tumor immune response that enhances the effects of immunotherapy [28, 29]. For this reason, we hypothesized that LPT could enhance the efficacy of immunotherapy. Since LPT increased the expression of markers of the lymphatic system in both young adult and aged mice, we assumed the effect was shared with the middle-aged mice who received LPT in the immunotherapy study and were not measured. However, this assumed increase in lymphangiogenesis induced by LPT did not enhance immunotherapy efficacy. One possible explanation is that lymphangiogenesis could be a net benefit for metastatic disease, and both LPT and immunotherapy can serve as distinct stimuli to elicit tumor lymphangiogenesis. This hypothesis would suggest that the impact of tumor lymphangiogenesis is stage-dependent, causing a detriment in primary disease by facilitating lymphatic metastasis but providing a net benefit in the treatment of metastatic disease by enhancing anti-tumor immunity.

To determine the effect of touch on the growth and spread of metastatic tumors, we employed an experimental metastasis model in which melanoma cells are injected systemically, resulting in metastatic tumors without a primary tumor. This approach reduces animal use and experimental duration while avoiding the well-established risk of mechanically prompting dissemination from a primary lesion. Taken together, this preclinical evaluation found that: (1) mechanical pressure generated by human touch affects metastatic and primary cancer differently, supporting an improved safety profile for therapeutic touch in patients with diffuse metastatic disease; (2) enhancing lymphatic flow with LPT decreased various measures of tumor metastasis in young adult and middle-aged animals, but not in those at an advanced age; (3) LPT increases the expression of lymphatic-specific markers in the tumor microenvironment; and (4) age is negatively correlated with LPT’s ability to mobilize white blood cells, red blood cells, and platelets into the circulation.

Several limitations temper the interpretation and clinical translation of these findings. This study relied on an experimental metastasis model that bypasses early stages of the metastatic cascade, including primary tumor formation, local invasion, and intravasation. These events could affect natural tumor behavior not accounted for in this study. In the first two experiments, LPT was administered to young adult and aged mice for 7 days before cancer cell injection, and every other day thereafter for 21 days. In the third experiment with middle-aged adult mice, LPT was administered only every other day after cancer cell injection. The intention of this approach was to simulate different clinical scenarios: 1) a patient receiving therapeutic touch before and after the metastatic event, and 2) a patient receiving therapeutic touch only after metastasis. Although we believe this increases the translatability of our findings, it is also a confounding mechanism. The observed effects of LPT in young adult and aged mice may have been caused by decreased seeding or increased clearance of circulating tumor cells rather than an anti-tumor immune response. Moreover, only one tumor type was evaluated, the B16F10 mouse melanoma cell line; this highly metastatic line is insufficient to draw conclusions about the behavior of human melanoma. Likewise, melanoma is a solid tumor type, so conclusions about blood-borne cancers cannot be drawn from this study. Accordingly, these results should be viewed as proof-of-concept within a constrained experimental framework, providing a foundation for future studies to more precisely define the safety and therapeutic potential of touch-based interventions in metastatic disease.

## Data Availability

The original contributions presented in the study are publicly available. This data can be found here [https://doi.org/10.6084/m9.figshare.31129240].
