# Supplementary material for: Lymphatic pumping technique in mice alters blood parameters and metastatic melanoma in an age-dependent manner
Source: Exp Biol Med (Maywood). 2026 Mar 30;251:10850. doi: 10.3389/ebm.2026.10850 (PMC13071501; doi:10.3389/ebm.2026.10850)
Supplement: Supplementary file 2 [file Supplementaryfile1.docx]

Supplemental File 1: Video of the lymphatic pumping technique used in the study. The video has been sped up by 5x.

Supplemental Figure 1: The effect of LPT on body composition parameters in middle-aged mice with metastatic melanoma.

*Changes in body composition from the beginning to the end of the experiment in each mouse were measured and analyzed using an ordinary one-way ANOVA with Tukey post hoc analysis. The bar graphs show the group mean, with 95% confidence intervals for the error bars. The compact letter display was included when the ANOVA showed a significant effect, and the post hoc analysis was conducted.*
