# Supplementary figures and images for: Lymphatic pumping technique in mice alters blood parameters and metastatic melanoma in an age-dependent manner
Source: Exp Biol Med (Maywood). 2026 Mar 30;251:10850. doi: 10.3389/ebm.2026.10850 (PMC13071501; doi:10.3389/ebm.2026.10850)

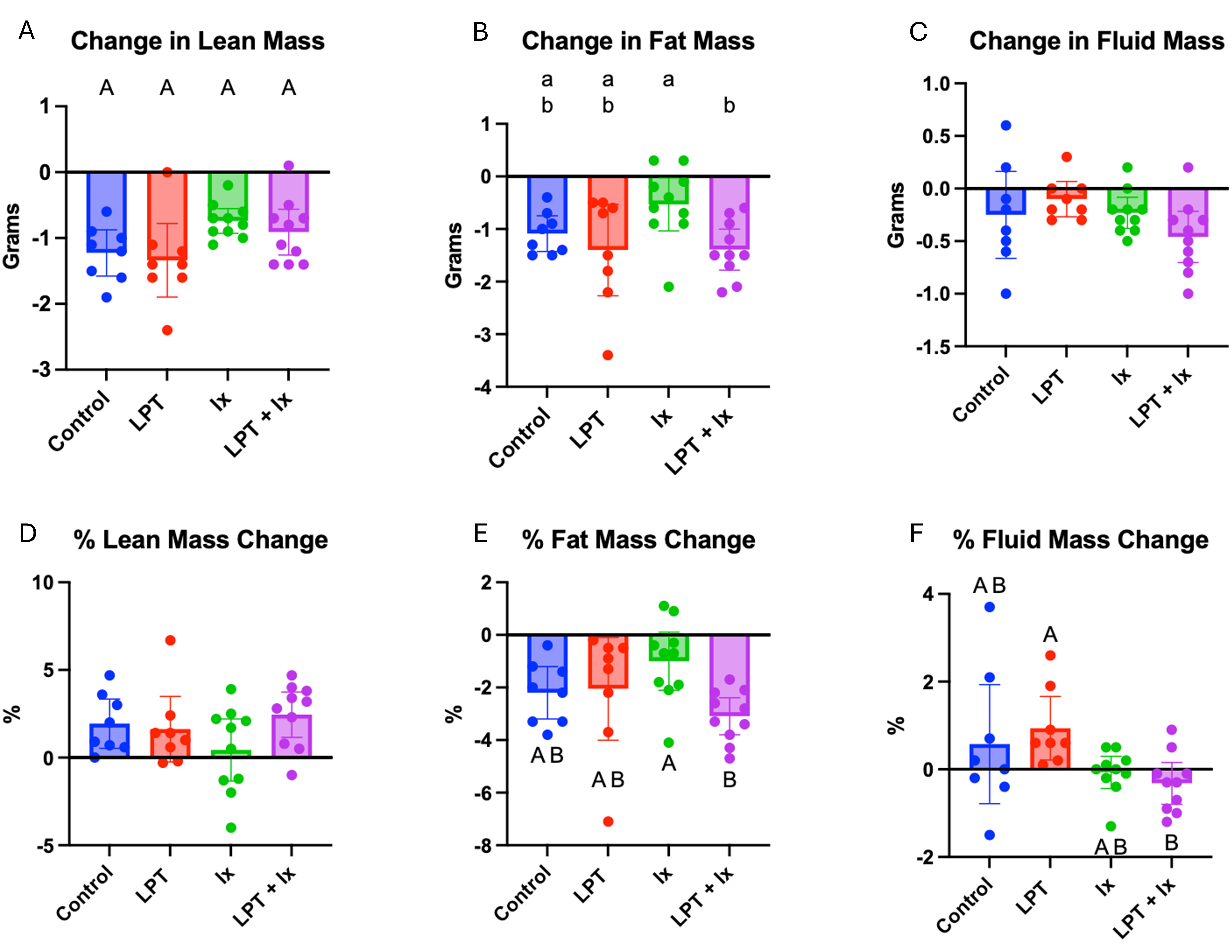

Supplement: Supplementary file 3 [file Image1.tif]
